# The brain’s best friend: microglial neurotoxicity revisited

**DOI:** 10.3389/fncel.2013.00071

**Published:** 2013-05-16

**Authors:** Sabine Hellwig, Annette Heinrich, Knut Biber

**Affiliations:** ^1^Department of Psychiatry and Psychotherapy, University Hospital FreiburgFreiburg, Germany; ^2^Department of Neuroscience, University Medical Center GroningenGroningen, Netherlands

**Keywords:** microglia, neuroprotection, mouse models, innate immunity, CX3CR1, microglia depletion

## Abstract

One long standing aspect of microglia biology was never questioned; their involvement in brain disease. Based on morphological changes (retracted processes and amoeboid shape) that inevitably occur in these cells in case of damage in the central nervous system, microglia in the diseased brain were called “activated.” Because “activated” microglia were always found in direct neighborhood to dead or dying neuron, and since it is known now for more than 20 years that cultured microglia release numerous factors that are able to kill neurons, microglia “activation” was often seen as a neurotoxic process. From an evolutionary point of view, however, it is difficult to understand why an important, mostly post-mitotic and highly vulnerable organ like the brain would host numerous potential killers. This review is aimed to critically reconsider the term microglia neurotoxicity and to discuss experimental problems around microglia biology, that often have led to the conclusion that microglia are neurotoxic cells.

## INTRODUCTION

Microglia research has intensified enormously in the last decade, and many surprising findings have been published. However, microglia still rank among the most mysterious cells of the brain and only recent results have begun to provide answers to the most basic questions in microglial biology, for example the origin of these cells, or the fact that microglia are not replaced by peripheral monocytes/macrophages in the healthy situation (Ajami et al., 2007; Ginhoux et al., 2010; Kierdorf et al., 2013). Moreover, it has become clear that “resting” microglia are by no means just idle cells (Nimmerjahn et al., 2005; Sierra et al., 2010; Tremblay et al., 2010; Paolicelli et al., 2011; Vinet et al., 2012) and there is good evidence to suggest that microglia are not only important in brain pathology but also play important roles in the healthy brain (Wake et al., 2009; Sierra et al., 2010; Tremblay et al., 2010; Paolicelli et al., 2011; Schafer et al., 2012). Thus, the general and simple concept of microglia “activation” is now questionable (see for recent reviews: Hanisch and Kettenmann, 2007; Colton, 2009; Ransohoff and Perry, 2009; Yong and Rivest, 2009; Graeber, 2010; Parkhurst and Gan, 2010; Ransohoff and Cardona, 2010; Kettenmann et al., 2011; Prinz et al., 2011; Tremblay et al., 2011; Aguzzi et al., 2013; Kettenmann et al., 2013). One long standing aspect of microglia biology, however, has never been challenged; namely, their involvement in brain disease, which was proposed many decades ago. This assumption was first based on morphological data, whereby ramified microglia in the healthy brain were described as “resting,” the rounded, macrophage-like microglia in the diseased brain were designated as “activated” microglia. Later it was shown that “activated” microglia sometimes express potential harmful substances, which led to the suggestion that these cells are detrimental during brain disease. On top of this, numerous cell culture experiments, most of which involved lipopolysaccharide (LPS)-treated microglia that have the potential to kill neurons, have further corroborated the assumption that “activated” microglia are neurotoxic cells (see for recent reviews: Block et al., 2007). However, from an evolutionary point of view it is difficult to understand why a highly sensitive, but otherwise long-lived, post-mitotic organ like the brain would serve as a host to such a large number of potentially toxic cells.

This review therefore aims to critically reconsider the common view that “activated” microglia are neurotoxic cells, and to highlight studies in which the role of microglia *in vivo* was specifically targeted, often revealing a protective function of these cells.

## MICROGLIA *IN VITRO* STUDIES

The first direct evidence concerning microglia as neurotoxic cells was published some 20 years ago (see for example: Boje and Arora, 1992; Chao et al., 1992). These experiments utilized standard microglia cultures (shake-off microglia from cultured neonatal brain homogenate) that were stimulated with rather high concentrations of single or combined pro-inflammatory stimuli such as LPS, interferon-gamma (IFN-γ), or tumor necrosis factor-α (TNF-α). These cells (or the resulting supernatant) were transferred to plates containing cultured neurons, and incubated for some time before neuronal survival was assessed (Boje and Arora, 1992; Chao et al., 1992). Ever since these pioneering experiments were performed, numerous variations of this experimental paradigm have identified a plethora of toxic microglial secretory products and/or detrimental microglia functions that obviously add weight to the notion that microglia are neurotoxic cells (see for recent examples: Lehnardt et al., 2008; Pais et al., 2008; Levesque et al., 2010; Burguillos et al., 2011; Gao et al., 2011). Thus, from the numerous papers that have investigated the influence of *in vitro* microglia on the survival of neurons, the majority has described a detrimental microglia role. Fewer studies have also found a neuroprotective function of cultured microglia showing that not all functions of cultured microglia are detrimental for neurons (see for recent review: Polazzi and Monti, 2010).

Cell culture experiments, however, should be approached with caution, especially when highly sensitive and reactive cells such as microglia are used. Standard cultured microglia have at least three major disadvantages: First, since standard cultured microglia are derived from the neonatal brain, these cells have missed the potential maturation process that occurs *in vivo*. Second, cultured microglia are grown in serum-containing (usually 10%) medium, whereas *in vivo* microglia normally never come in contact with serum components. Third, nowadays it is also very well known that *in vivo* microglia are kept under constant restraint by a variety inhibitory inputs such as CX3CL1, CD200, CD22, or CD172 (see for review: Biber et al., 2007; Ransohoff and Cardona, 2010; Prinz et al., 2011), which, of course, is not the case in culture. Indeed, the genetic removal of even just one of these inhibitory factors in animal models dramatically changes the reaction profile of microglia, often causing overshooting microglia reactions and sometimes even toxic microglia responses (Hoek et al., 2000; Cardona et al., 2006); therefore, it is very likely that the complete lack of normal inhibition has a dramatic influence on the reactivity of cultured microglia.

Despite the caveats associated with studying microglial function *in vitro*, there is surprisingly little research regarding the question of whether cultured microglia can be reliably compared to their *in vivo* counterparts. One such report by Boucsein et al. (2000) investigated the electrophysiological properties of microglia by comparing cultured (with or without LPS treatment) and ramified microglia in acute brain slice preparations. It was found that ramified microglia barely display membrane currents, in stark contrast to primary cultured microglia, which elicited inward and outward rectifying currents (depending on LPS treatment) that were similar to those found in cultured macrophages (Boucsein et al., 2000). More recently, Schmid et al., 2009 compared mRNA expression profiles between cultured microglia and alveolar macrophages stimulated with LPS/IFN-γ and microglia rapidly isolated from the brain of LPS/IFN-γ treated animals. This study also reported that cultured microglia and macrophages are much more alike than the microglia that have been acutely derived from brain tissue (Schmid et al., 2009). Recently, a similar comparative analysis was performed for post-mortem human microglia and macrophages derived from the choroid plexus (Melief et al., 2012). These authors not only provided convincing evidence for major differences in surface marker and mRNA expression pattern between brain-derived microglia and macrophages, they further showed that acutely isolated microglia are not able to respond to LPS stimulation, most likely because these cells lack CD14 (Melief et al., 2012). It is yet not known whether this lack of CD14 and LPS response is due to the isolation technique used in the study. However, overnight incubation in culture increased CD14 levels and rendered the cells sensitive to LPS treatment, again suggesting that growing microglia in culture can have a tremendous influence on the reactivity of these cells (Melief et al., 2012). These results strongly implicate that cultured microglia share few similarities with their *in vivo* counterparts, which leads to the conclusion that *in vitro* evidence concerning microglia should be interpreted with the utmost caution when extrapolating data into the context of the brain.

## NEURONAL LOSS AND THE PRESENCE OF AMOEBOID MICROGLIA: CHICKEN OR EGG?

Histological studies by Del Rio-Hortega identified microglia almost a century ago (see Kettenmann et al., 2011 for an excellent overview on microglia history) and even back in the early days of microglia research, the potential importance of these cells in brain disease was already been recognized. Indeed, these seminal histological studies on microglia morphology also gave rise to the concept of microglia “activation,” which states that ramified microglia in the healthy brain are in a resting state, and that upon any potential danger signal these cells morph into an amoeboid or macrophage-like shape. Because the complexity of this morphological transition is limited, microglia responses were generally seen as graded and stereotypic (see for review: Kettenmann et al., 2011). In other words it was more or less believed that microglia always had the same role once they become amoeboid. Numerous *in vivo* reports have concluded that amoeboid microglia can potentially confer neurotoxicity by expressing substances (that are toxic *in vitro*) such as pro-inflammatory cytokines (Blais and Rivest, 2004; Xie et al., 2004; Allan et al., 2005; Kim and de Vellis, 2005; Walker and Lue, 2005). Other reports used minocycline (a potential microglia inhibitor) or other anti-inflammatory drugs to ameliorate damage in different models of brain diseases and since at the same time a decreased morphological transition of microglia was observed these results were often discussed in favor of a neurotoxic role of amoeboid microglia (see for example: Yrjänheikki et al., 1998; He et al., 2001; Tikka et al., 2001; Kriz et al., 2002; Hunter et al., 2004; Fan et al., 2005).

However, it could very well be that the morphological transition of microglia is the result rather than the cause of neuronal damage, as was shown in a model of 1-methyl-4-phenyl-1,2,3,6-tetrahydropyridine (MPTP) and methamphetamine (METH)-induced neurotoxicity (O’Callaghan et al., 2008). In addition, the fact that microglia may undergo morphological transition in the absence neuronal loss is often disregarded. For example, although the injection of LPS *in vivo *causes rapid morphological transition of microglia, yet it does not induce massive neuronal death, except when injected into the substantia nigra (Kim et al., 2000; Nadeau and Rivest, 2002, 2003). Moreover, it has been known for more than 10 years that microglia in the spinal cord become amoeboid in response to peripheral nerve injury, which is important for the development of neuropathic pain. However, despite the presence of activated microglia, neuronal loss is not a hallmark of neuropathic pain.

Taken together, we think that there is a bias in the field regarding the function of amoeboid microglia. The temporal and spatial relationship between amoeboid microglia-like cells (see next chapters) and dead or dying neurons -which inevitably occurs in case of neuronal damage- is often seen as indication for a neurotoxic role of microglia. Such correlation, of course, does not allow conclusions about causality.

## TARGETING MICROGLIA IN MOUSE MODELS OF DISEASE

Using an experimental approach to understand microglial function is a challenging task and as a result most studies, although carefully performed, did not specifically target microglia. In numerous studies is a microglia reaction induced by exogenous application of pro-inflammatory molecules (injection of IL-1, TNFα, LPS, and others) that unleash an uncontrollable immune response, not only in the brain (which also potentially involves astrocytes, oligodendrocytes, endothelial cells, perivascular macrophages, and neurons), but also most likely causes an immune response in the periphery. The second-generation tetracycline minocycline is often referred to as a specific inhibitor of microglia (see for example: Raghavendra et al., 2003; Ledeboer et al., 2005; Mika et al., 2007; Osikowicz et al., 2009). However, it should be kept in mind that minocycline is much less specific than often stated, as it clearly affects peripheral macrophages (Dunston et al., 2011), and T cells (Szeto et al., 2011), and can have a direct influence on the survival of neuronal cell lines and cultured neurons (Hashimoto and Ishima, 2010; Huang et al., 2010; Schildknecht et al., 2011; Ossola et al., 2012), thus, numerous microglia-independent effects of minocycline have been published (Hughes et al., 2004). Moreover, it was reported that the microglia reaction in response to facial nerve injury is unchanged in the presence of even high concentrations of minocycline (Fendrick et al., 2005). Similarly, other anti-inflammatory drugs are not specific for microglia either; for example, the neuroprotective effect of the anti-inflammatory compound triflusal was found not to depend on the presence of microglia (Montero Domínguez et al., 2009). It can thus be concluded that more specific methods are needed to address microglia function.

## MICROGLIA VS. OTHER PERIPHERAL MONOCYTES/MACROPHAGES

### THE PROBLEM OF IDENTIFICATION

Microglia are derived from early myeloid precursor cells that appear in the yolk sac before major vascularization or hematopoiesis occurs in the developing embryo (Sorokin et al., 1992; Alliot et al., 1999; Herbomel et al., 2001; Ginhoux et al., 2010; Mizutani et al., 2012). Strikingly, it was found that microglia stem from primitive erythromyeloid progenitor cells that develop via a special program into mature microglia, and most importantly that these cells form a stable self-contained population that is not replaced by peripheral monocytes in the unchallenged brain (Kierdorf et al., 2013; Neumann and Wekerle, 2013).

While microglia can be easily identified in the healthy brain, this changes under pathological conditions in which peripheral monocytes/macrophages enter the brain. Despite the fact that microglia and peripheral monocytes/macrophages have different developmental origins, both cell populations share many properties. The expression of general innate pattern recognition receptors such as Toll-like receptors (TLR), nucleotide-binding oligomerization domain (NOD)-lime receptors (NLR), or complement receptors are common to both microglia and peripheral monocytes/macrophages, as is the ability to secrete a whole variety of different cytokines (pro- and anti-inflammatory), growth factors, chemokines, reactive oxygen, and nitrogen species (Kettenmann et al., 2011; Jung and Schwartz, 2012). The lack of reliable microglia-specific markers makes it very difficult both to discriminate between microglia and peripheral monocytes/macrophages, to allocate functions to either cell type (Jung and Schwartz, 2012). This difficulty may have added confusion to the question whether or not microglia in the diseased brain are neurotoxic cells: an example here is the chemokine receptor CCR2. On one hand there are various reports in which CCR2 expressing cells are suggested to be microglia (Abbadie et al., 2003; Zhang et al., 2007; Fernández-López et al., 2012) or described as microglia/macrophages (Yao and Tsirka, 2012) or referred to as amoeboid microglia cells (Deng et al., 2009). Often CCR2 is discussed to be an important receptor for the recruitment of microglia to injured brain areas (El Khoury et al., 2007; Zhang et al., 2007; Deng et al., 2009; Raber et al., 2013) and the inhibition or lack of CCR2 signaling is related to improved disease outcome (Abbadie et al., 2003; Dimitrijevic et al., 2007; Zhang et al., 2007; Fernández-López et al., 2012; Yao and Tsirka, 2012) implicating that CCR2-expressing microglia at least contribute to disease progression.

On the other hand there is convincing evidence from different transgenic mouse models and bone-marrow transplantation experiments that microglia do not express CCR2 in the healthy or diseased brain (Mildner et al., 2007; Jung et al., 2009; Saederup et al., 2010; Mizutani et al., 2012), moreover mouse microglia lack the mRNA for CCR2 (Zuurman et al., 2003; Olah et al., 2012). In bone-marrow transplantation experiments it was shown that the response of endogenous microglia to stroke was not affected in CCR2 deficient animals, showing that CCR2 is not regulating microglia responses here (Schilling et al., 2009a,b). In these studies it was shown that the infiltration of peripheral monocytes into the brain was greatly reduced, which is in agreement with other reports clearly showing that peripheral monocytes require CCR2 in order to invade the diseased central nervous system (CNS; Mildner et al., 2007; Schilling et al., 2009a,b; Prinz and Priller, 2010; Prinz and Mildner, 2011; Mizutani et al., 2012).

Thus CCR2 in the brain should be regarded as marker of peripheral monocytes/macrophages making it questionable whether the published (mostly detrimental) effects of CCR2 expressing cells can be allocated to microglia. CCR2 most likely is not the only example in this respect, showing that without a proper identification it is not possible to draw conclusions about microglia function.

### HOW TO TELL THEM APART?

One way to discriminate between monocytes/macrophages and microglia is offered by flow cytometry analysis [(fluorescence assisted cell sorting (FACS)] of acutely isolated cell preparations from the diseased brain using CD11b and CD45 antibodies. Although monocytes/macrophages and microglia are both positive for CD11b and CD45, microglia can be identified by their relatively low expression levels of CD45 (“CD45dim”) compared to those of peripheral monocytes/macrophages (“CD45high”). Thus microglia and monocytes/macrophages appear as separate cell populations in FACS analysis (Sedgwick et al., 1998; de Haas et al., 2007, 2008; Remington et al., 2007).

We used such a FACS-based identification approach in a mouse model of cuprizone-induced loss of oligodendrocytes, whereby microglia were specifically isolated from the corpus callosum of mice before cuprizone treatment, during demyelination and after remyelination (Olah et al., 2012). Thus, pure preparations of ramified microglia (healthy controls), amoeboid microglia (peak of demyelination) and microglia returning to the quiescent state (2 weeks post-remyelination) were subjected to genome-wide gene expression analysis. One aim of this study was to describe the two-sides of microglia activity, based on our expectation of finding a pro-inflammatory profile in the amoeboid microglia isolated during the demyelination phase, and a more anti-inflammatory expression profile in microglia isolated from the corpus callosum during the remyelination period. To our surprise, we did not find any evidence for a double-edged function of microglia during the disease course in this model. Instead we observed a microglial phenotype that supported remyelination at the onset of demyelination, a function which persisted throughout the remyelination process (Olah et al., 2012). Our data showed that microglia are involved in the phagocytosis of myelin debris and apoptotic cells during demyelination (Olah et al., 2012). Furthermore, microglia displayed cytokine and chemokine expression profiles that were associated with the activation and recruitment of endogenous oligodendrocyte precursor cells to the lesion site, as well as the delivery of trophic support during remyelination (Olah et al., 2012). In other words, although corpus callosal microglia displayed an amoeboid morphology under demyelinating conditions, these cells expressed proteins that, rather than potentially contributing to oligodendrocyte death, actually initiated a repair response, even during the early onset phase of the disease.

Another way to discriminate peripheral monocytes/macro-phages vs. endogenous microglia are bone-marrow transplantation or parabiosis experiments. Both have been utilized in experimental allergic encephalomyelitis (EAE) and it was observed that peripheral myeloid cells utilize CCR2 in order to invade the diseased brain and that an inhibition of this invasion resulted in a significantly diminished EAE disease course, leading the authors to conclude that peripheral monocytes/macrophages but not resident microglia cells are responsible for disease progression in EAE (Mildner et al., 2009; Ajami et al., 2011). Using bone-marrow transplantation and different genetic models it was further demonstrated that in acute spinal cord injury, endogenous microglia also differ functionally from invading peripheral monocytes/macrophages (see for review: Jung and Schwartz, 2012).

Thus there are various studies that convincingly demonstrate that peripheral monocytes/macrophages may have significantly different functions compared to endogenous microglia in the diseased brain. Interestingly, a more specific analysis of microglia (as discussed above) often (but not always, see Jung and Schwartz, 2012) revealed beneficial, or at least non-toxic microglia responses.

## LACK OF CX3CR1 OR CD200r IN MICROGLIA

As mentioned above, nowadays it is clear that microglia in the brain are under constant restraint, particularly because they specifically express receptors for a variety of inhibitory factors that are constitutively expressed in the brain, mostly by neurons (Biber et al., 2007; Ransohoff and Perry, 2009). The most prominent ligand-receptor pairs in this respect are CX3CL1-CX3CR1 and CD200-CD200r. Regarding the CX3CR1-CX3CL1 axis, one of the most used mouse model in microglia research is the CX3CR1-EGFP mouse line in which all microglia are green fluorescent protein (GFP)-positive (Jung et al., 2000). This mouse line, has since contributed enormously to our current understanding of microglia biology, and CX3CR1-deficient homozygotes have been used extensively to study the role of CX3CR1 in various models of brain disease. Indeed, the consequences of CX3CR1 deletion in microglia largely depends on the mouse model used (see for extensive review: Prinz et al., 2011; Ransohoff and Prinz, 2013; Wolf et al., 2013); however, the overall idea at the moment is that a lack of CX3CR1 leads to the “hyperactivity” of microglia in the diseased brain, thereby unleashing potential neurotoxic properties (Wolf et al., 2013). Accordingly, administration of CX3CL1 into the brain causes neuroprotection in experimental stroke and two models of Parkinsons disease (Cipriani et al., 2011; Pabon et al., 2011; Morganti et al., 2012) Similarly, removing the inhibitory input that is normally modulated by CD200 [i.e., as in CD200r knockout (KO) mice] reportedly promotes microglial morphological transition even in the healthy brain (Hoek et al., 2000) and leads to an exaggerated disease course both in EAE (Broderick et al., 2002) and retinal inflammation (Hoek et al., 2000).

What remains to be established is the question whether the CX3CL1-CX3CR1 or CD200-CD200r axes are affected in the diseased brain. To this end, there are only a few reports about CXC3L1 levels in the brain and CX3CR1 expression levels in microglia during the course of disease. For example, Cardona et al. (2006) detected rather high levels of free CX3CL1 in the brain (around 300pg/mg), which is suggestive of constitutive CX3CL1 release under normal physiological conditions. In the diseased (rodent or human) brain, the levels of CX3CL1 and/or CX3CR1 were found either to be unchanged or increased (Hughes et al., 2002; Tarozzo et al., 2002; Hulshof et al., 2003; Lindia et al., 2005; Xu et al., 2012), indicating that the inhibitory function of the CX3CL1-CX3CR1 axis is not generally weakened under diseased conditions. This might be different in the aged brain or brains of Alzheimer patients where a downregulation of CX3CL1 was recently observed (Wynne et al., 2010; Cho et al., 2011).

Even less is known about the regulation of CD200 or CD200r expression in disease. It was reported that in human multiple sclerosis (MS) patients, CD200 expression in neurons diminishes around the periphery and in the center of MS lesions (Koning et al., 2007); however, astrocytes in these lesions acquire CD200 expression (Koning et al., 2009). In a mouse model of hippocampal excitotoxicity, an increase in neuronal CD200 expression was observed (Yi et al., 2012), while a decrease in CD200 and CD200r expression was reported in a mouse model of Alzheimer’s disease (Walker et al., 2009)

Thus, it is at the moment unclear whether CX3CR1 or CD200r signaling is diminished in the diseased brain. To gain more knowledge about the regulation of the microglia inhibitory environment during brain disease is, however, of importance for our interpretation of the results gained in mice with mutated CX3CL1-CX3CR1 or CD200-CD200r signaling. In other words it remains to be established whether or not in a given brain disease the inhibitory input for microglia is decreased and as a result these cells become “hyperactivated” and potentially neurotoxic.

## OTHER MOUSE MODELS WITH MUTATED MICROGLIA (FUNCTION)

In amyotrophic lateral sclerosis (ALS), mutations within the ubiquitously expressed enzyme superoxide dismutase 1 (SOD1) gene are responsible for about a quarter of the inherited disease cases. Accordingly, mice that express mutant human SOD1 exhibit motoneuron degeneration and a decreased life span (see for review: Lobsiger and Cleveland, 2007). The role of microglia in this disease has been investigated in various elegant experiments in which mutated SOD1 was expressed in specific cell types (Pramatarova et al., 2001; Lino et al., 2002; Clement et al., 2003; Beers et al., 2006; Boillée et al., 2006). The conclusion that arose from these experiments was that microglia with mutated SOD1 do not initiate motor neuron degeneration but rather accelerate disease progression (see for review: Lobsiger and Cleveland, 2007), since the replacement of SOD1 mutated microglia with wild-type cells slowed down disease progression and prolonged the life span of the animals (Beers et al., 2006); this effect required functional MyD88 signaling in microglia, indicating that proper immune function of these cells is necessary in order to inhibit disease progression and prolong the lifespan of animals (Kang and Rivest, 2007). Similarly, it was recently reported that transplantation of wild-type microglia into the brains of mice deficient for methyl-CpG binding protein (MECP2-/-; a model for Rett syndrome) ameliorated disease progression and significantly increased the life span of the animals (Derecki et al., 2012).

## MUTATED HUMAN MICROGLIA

Triggering receptor expressed on myeloid cells-2 (TREM2) is another receptor that is in brain exclusively expressed in microglia (for review see: Linnartz et al., 2010). TREM2 belongs to the family of immunoreceptor tyrosine-based activation motif (ITAM) receptors for which the ligand has yet not been identified. Activation of TREM2 stimulates phagocytic activity in microglia and downregulates TNFα and inducible nitric oxide synthase (iNOS) expression (Takahashi et al., 2005). TREM2 is thus an anti-inflammatory receptor that at the same time promotes phagocytic activity. TREM2 is intracellularly coupled to the adapter protein DAP12 (see for review: Linnartz et al., 2010), and interestingly, loss of function mutations of either TREM2 or DAP12 lead to a rare chronic neurodegenerative disease known as Nasu–Hakola or polycystic lipomembranous osteodysplasia with sclerosing leukoencephalopathy (PLOSL), an inherited autosomal recessive human disease characterized by early onset presenile dementia (Colonna, 2003).

It can thus be appreciated from the studies discussed in the last three chapters that milder disease or less neuronal loss in the presence of mutated microglia is seldom occurring. In contrary, the perturbation of proper microglia function by various mutations regularly leads to neuronal dysfunction and/or neurodegeneration, findings that would not corroborate the idea that microglia can become neurotoxic cells. It should be noted that the problem of microglia vs. peripheral monocytes/macrophages, however, also is of importance here since none of the above described mutated genes is exclusively found in microglia.

## MODELS OF MICROGLIAL INHIBITION OR DEPLETION

### MICROGLIA DEPLETION WITH CLODRONATE

The bisphosphonate drug clodronate is toxic to cells of the myeloid lineage and can be used to selectively deplete them *in vivo* and *in vitro* (Buiting and Van Rooijen, 1994). Since microglia are of myeloid origin, clodronate can also be used to deplete microglia in cell culture, organotypic hippocampal slice cultures (OHSC) and *in vivo* (Kohl et al., 2003; Lauro et al., 2010; Drabek et al., 2012). OHSC are *in vitro* explant cultures that reflect many aspects of the hippocampus *in vivo* situation by maintaining a certain degree of intrinsic connectivity and lamination (see for example: Frotscher et al., 1995). With respect to microglia, it is known that after 10 days in culture these cells acquire a ramified morphology that is comparable to their *in vivo* counterparts (Hailer et al., 1996). OHSC neurons of the CA1, CA3, and DG regions display distinct and selective neuronal vulnerability toward *N*-methyl-D-aspartate (NMDA)-induced excitotoxicity, with CA1 neurons being the most susceptible, followed by CA3 and DG neurons, respectively (Vornov et al., 1991; Cronberg et al., 2005; Boscia et al., 2006; Gee et al., 2006). Importantly, this effect has also been observed *in vivo* (Kirino and Sano, 1984; Horn and Schlote, 1992; Acarin et al., 1996; Won et al., 1999; Schauwecker, 2002). In addition, there is a strict correlation between the amount of neuronal loss occurring and the morphological profile of microglia in OHSC subjected to excitotoxicity (Heppner et al., 1998; van Weering et al., 2011; Vinet et al., 2012). We have used OHSC to address the function of microglia in NMDA-induced neuronal loss by depleting microglia and then replenishing them with microglia (Vinet et al., 2012). It was found that neuronal cell loss was prominently increased in the absence of microglia and that even neurons of the DG were affected by the NMDA treatment when microglia cells were not present (Vinet et al., 2012). These findings are in agreement with earlier reports from our group, as well as from others (Montero et al., 2009; van Weering et al., 2011). In addition to earlier findings, we also showed that when microglia-free OHSCs were replenished with microglia, these cells invaded the tissue, distributed themselves evenly across the slice and acquired an *in vivo*-like, ramified morphology (Vinet et al., 2012). Most importantly, neurons in the presence of these ectopic microglia were protected from NMDA-induced toxicity to the same extent as in non-depleted control slices (Vinet et al., 2012). These findings convincingly show not only that microglia have a neuroprotective capacity, but also that this property applies to ramified microglia (Vinet et al., 2012). Thus, neurons are protected in the vicinity of ramified microglia, while removing microglia from the local environment renders neurons more vulnerable to excitotoxicity.

Although it is yet not clear whether similar processes also occur *in vivo*, it is tempting to speculate that numerous protective properties of microglia have simply gone unnoticed because ramified microglia were generally long considered to be inactive cells. This speculation is corroborated by recent findings in the neonatal brain subjected to middle cerebral artery occlusion (MCAO), a widely used stroke model. Here it was reported by Faustino et al. (2011) that the depletion (or reduction) of ramified microglia *in vivo* (by intracerebral injection of clodronate-filled liposomes) exacerbated injury after stoke. Interestingly, the initial effect of the MCAO did not change in the absence of microglia, however, the volume of the lesion gradually increased over time (Faustino et al., 2011), again suggesting that the absence of ramified microglia is detrimental to the injured brain.

### CD11b HSVTK MOUSE LINES

Another way to specifically target microglia is through the use of transgenic mouse strains in which the herpes simplex virus thymidine kinase (HSVTK) is placed under the control of the CD11b promoter (Heppner et al., 2005; Gowing et al., 2006). Treating OHSCs from these animals with ganciclovir efficiently depletes microglia from the tissue slice culture (Falsig et al., 2008; Vinet et al., 2012). As a result the lack of microglia increases prion titers by 15 times and thereby also the susceptibility of the slices to prion infection was enhanced (Falsig et al., 2008). From these results the authors concluded that microglia are important elements in containment of prion infections (Falsig et al., 2008).

Application of ganciclovir to CD11b-HSVTK animals leads to the death of proliferating CD11b+ cells (Heppner et al., 2005; Gowing et al., 2006). The effects of ganciclovir on microglia *in vivo* are dependent on the application route of the drug in these animals. If peripheral ganciclovir application (intraperitoneal injection or oral application) is used, transplantation of wild-type bone-marrow is required to spare the peripheral myeloid compartment from ganciclovir treatment. In these resulting chimeric animals, ganciclovir application leads to the inhibition of morphological microglia transition to amoeboid cells in the case of EAE (referred to as microglia paralysis Heppner et al., 2005), or to the death of microglia undergoing proliferation after experimental stroke (Lalancette-Hébert et al., 2007). No effects of peripheral ganciclovir application on ramified microglia were found in these studies (Heppner et al., 2005; Lalancette-Hébert et al., 2007). Whereas the inhibition of morphological microglia transition (microglia paralysis) was protective in EAE (delayed disease onset and reduced clinical scores; Heppner et al., 2005), the ablation of microglia proliferation in the stroke model led to a larger stroke lesion area and increased neuronal death (Lalancette-Hébert et al., 2007).

More recent studies using these mouse lines have changed the application route of ganciclovir from peripheral to central, which has a twofold benefit. First, the need for bone-marrow transplantation is circumvented, and second ramified microglia are also sensitive to centrally delivered ganciclovir, which depletes the treated brain tissue of ramified microglia (Gowing et al., 2008; Grathwohl et al., 2009; Mirrione et al., 2010; Varvel et al., 2012). In the corresponding studies it was shown that the depletion of microglia by ganciclovir did not affect the development of beta amyloid plaques in two different mouse models of Alzheimer’s disease (Grathwohl et al., 2009), nor did the absence of microglia change disease progression and motor neuron degeneration in the SOD mouse model of ALS (Gowing et al., 2008). However, in the case of pilocarpine-induced seizures, the depletion of microglia prevented the protective effect of LPS pre-conditioning, indicating that the inflammatory capacity of microglia is beneficial in this mouse model (Mirrione et al., 2010). Taken together, it can be concluded that ganciclovir-dependent inhibition of microglia function in CD11b-HSVTK animals was only advantageous in a disease model in one reported case (Heppner et al., 2005). All other reports either provided evidence for a beneficial role of microglial function *in vivo* (Lalancette-Hébert et al., 2007; Mirrione et al., 2010) or showed no effect of blunting the microglial response (Gowing et al., 2008; Grathwohl et al., 2009). It should be noted here that the latter studies inhibited or depleted microglia for a limited time at rather late stages of chronic disease models (Gowing et al., 2008; Grathwohl et al., 2009), which may explain the surprising lack of effect. The inhibition or depletion of microglial function may have been too late or too short to unravel the role of these cells in mouse models of AD and ALS (Gowing et al., 2008; Grathwohl et al., 2009). Thus, inhibition or depletion of microglia for longer time periods may be required for chronic disease models.

### CD11b-DTR MICE

Another mouse model that allows the depletion of myeloid cells is the CD11b-DTR mouse line that expresses diphtheria toxin receptor under the control of the CD11b promoter (Duffield et al., 2005). This mouse was very recently used to study the role of microglia in the development of the cortex (Ueno et al., 2013). This study shows that impaired microglia function or depletion of these cells by diphtheria toxin injection leads to enhanced neuronal loss of layer V neurons. The authors furthermore provide evidence that microglia provide trophic support for layer V neurons through the synthesis and release of IGF1. Interestingly, this vital role of microglia for layer V neurons was attributed to amoeboid microglia (Ueno et al., 2013).

Taken together, depleting microglia is rarely correlated to improved outcome in various brain disease models. These findings are thus not in favor of a major neurotoxic function of microglia in the brain but would argue more for a protective role of the innate immune cells of the brain.

## CONCLUSION

Innate immunity was originally seen as a stereotypic response to exogenous pathogens. However, the “danger model” formulated by Polly Matzinger more than a decade ago (Matzinger, 2002) couple with more recent findings that pattern recognition receptors are also activated by endogenous ligands [so-called danger-associated molecular patterns (DAMP)] have dramatically challenged this original view (Gordon, 2002). Even the classical immune defense components, such as the complement system, which are activated to ward off pathogens have now been recognized as danger signals that are not necessarily linked to pathogen infection (Köhl, 2006). Moreover, innate immune cells are no longer considered to elicit a stereotypic response. In striking contrast, it now is apparent that in the face of different kinds of threats, innate immune cells are able to interpret signals and launch an appropriate response (see for review: Gordon, 2003; Martinez et al., 2008; Mosser and Edwards, 2008; Gordon and Mantovani, 2011). Hence, it is now clear that tissue damage or cellular stress is also a potent inducer of innate immunity, where the ultimate goal is to protect and restore cellular function, thereby guaranteeing the functional integrity of the body (Medzhitov, 2010). Microglia, the innate immune cells of the brain, should be viewed along the same lines, because infections with exogenous pathogens are (luckily) scarce events in the brain making cellular stress or tissue damage more likely signals for microglia.

In this review we argue that amoeboid microglia have a bad reputation. Based on correlative histological data and corresponding *in vitro* experiments these cells are often discussed to be neurotoxic. Moreover, their striking similarity with peripheral monocytes/macrophages has blurred our picture concerning the function of these cells. Microglia colonize the CNS very early and are sequestered there throughout a lifetime, making it very likely that these cells have an elaborate repertoire of brain specific functions that may not be appropriately taken over by peripheral monocytes/macrophages (Neumann and Wekerle, 2013). Therefore to discriminate microglia from peripheral monocytes/macrophages and to elucidate the functional spectrum of microglia in the (healthy and diseased) brain will be a major challenge.

New mouse models may be helpful here. It was recently shown that following chemical depletion of microglia (CD11b-HSVTK mouse) there is a rapid and efficient repopulation of the brain with CD45 high and CCR2+ blood monocytes, which gradually engrafted into the microglia-free tissue with an overall distribution and morphology reminiscent (yet different) to that of endogenous microglia (Varvel et al., 2012). This mouse model thus offers an opportunity to investigate the question whether or not invaded monocytes/macrophages can become true brain microglia (Varvel et al., 2012; Neumann and Wekerle, 2013). Other new mouse models may also be helpful to distinguish microglia (function) from peripheral monocytes macrophages. The double knock-in mouse expressing red fluorescent protein (RFP) and GFP in the CCR2 and CX3CR1 locus, respectively, allows a reliable discrimination of both cell types even in tissue sections (Mizutani et al., 2012), and the newly described CX3CR1^creER^-line can be utilized to construct KO or overexpression models that will enable us to investigate the *in vivo* functions of receptors, signaltransduction pathways or inflammatory mediators in a microglia specific context (Yona et al., 2013; Wolf et al., 2013).

Using such models we may now be able to investigate the overall picture of microglia responses, for example in the development of neuropathic pain. There is compelling evidence that amoeboid microglia in the spinal cord release brain-derived neurotrophic factor (BDNF) in response to peripheral nerve lesion, which is crucial for the development of neuropathic pain (Tsuda et al., 2003; Coull et al., 2005; Ulmann et al., 2008). Accordingly, microglia are seen as inducers of a pathological pain reaction, again indicating that their action in response to nervous damage is detrimental (see for recent review: Tsuda et al., 2013). The whole functional spectrum of microglia derived BDNF under these circumstances, however, is not known at the moment. Since BDNF is a neuronal survival factor it is tempting to speculate that one reason for its release from microglia is to protect neurons from nerve injury. It may therefore be of interest to analyze the neuronal fate after peripheral nerve injury in microglia-specific BDNF KO animals.

Taken together, mutating or deleting microglia very often leads to the development or worsening of a brain disease, results that are favoring a beneficial role of these cells in the CNS. It is discussed here that several experimental problems around these cells have led to potential misinterpretations concerning the role of microglia. It is thus clear that without (i) a detailed analysis of the causal relationship between microglial function and neuronal fate, and (ii) a thorough understanding of all aspects of the microglia response in a given disease (model), it is difficult to fully appreciate what microglia are doing in the brain. As new techniques and mouse models are now emerging to do such analysis, it is anticipated that more surprising findings about “the brain’s best friend” will be published.

## Conflict of Interest Statement

The authors declare that the research was conducted in the absence of any commercial or financial relationships that could be construed as a potential conflict of interest.
